# Digital breast tomosynthesis versus X-ray of the breast specimen for intraoperative margin assessment: A randomized trial

**DOI:** 10.1016/j.breast.2023.103616

**Published:** 2023-12-01

**Authors:** Irina Palimaru Manhoobi, Trine Tramm, Søren Redsted, Anne Bodilsen, Leslie Foldager, Peer Christiansen

**Affiliations:** a Department of Radiology, Aarhus University Hospital, Aarhus, Denmark; b Department of Pathology, Aarhus University Hospital, Aarhus, Denmark; c Department of Abdominal Surgery, Aarhus University Hospital, Aarhus, Denmark; d Department of Animal and Veterinary Sciences, Aarhus University, Tjele, Denmark; e Bioinformatics Research Centre, Aarhus University, Aarhus, Denmark; f Department of Plastic- and Breast Surgery, Aarhus University Hospital, Denmark; g Department of Clinical Medicine, Aarhus University, Aarhus, Denmark

## Abstract

**Background:**

Involved resection margins after breast conserving surgery (BCS) often require a re-operation with increased patient anxiety and risk of impaired cosmesis.

We investigated the number of re-operations due to involved resection margins after BCS comparing digital breast tomosynthesis(DBT) with X-ray for intraoperative margin evaluation.

Furthermore, we assessed the diagnostic accuracy of these methods to predict histopathological margin status. Finally, we evaluated risk factors for re-operation.

**Methods:**

In this randomized, non-blinded study, 250 invasive breast cancer patients were randomized (1:1), whereof 241 were analyzed intraoperatively with either DBT (intervention, n = 119) or X-ray (standard, n = 122). Pearson's chi-squared test, Fisher's exact test, *t*-test, logistic and ordinal regression analysis was used as appropriate.

**Results:**

No difference was found in the number of re-operations between the DBT and X-ray group (16.8 % vs 19.7 %, p = 0.57), or in diagnostic accuracy to predict histopathological margin status (77.5 %, CI: 68.6–84.9 %) and (67.3 %, CI: 57.7–75.9 %), respectively. We evaluated 5 potential risk factors for re-operation: Ductal carcinoma in situ (DCIS) outside tumor, OR = 9.4 (CI: 4.3–20.6, *p* < 0.001); high mammographic breast density, OR = 6.1 (CI: 1.0–38.1, *p* = 0.047); non-evaluable margins on imaging, OR = 3.8 (CI: 1.3–10.8, *p* = 0.016); neoadjuvant chemotherapy, OR = 3.0 (CI: 1.0–8.8, *p* = 0.048); and T2 tumor-size, OR = 2.6 (CI: 1.0–6.4, *p* = 0.045).

**Conclusions:**

No difference was found in the number of re-operations or in diagnostic accuracy to predict histopathological margin status between DBT and X-ray groups. DCIS outside the tumor showed the highest risk of re-operation. Intraoperative methods with improved visualization of DCIS are needed to obtain tumor free margins in BCS.

## Highlights

• No difference in re-operations between using digital breast tomosynthesis and specimen X-ray in breast conserving surgery.
• Ductal Carcinoma in Situ (DCIS) in the resected margins was strongly associated with re-operation.
• Improved imaging of DCIS during breast conserving surgery is needed to obtain tumor free margins.

## 1 Introduction

Breast conserving surgery (BCS) followed by whole breast irradiation has become the standard treatment of choice in early invasive breast cancer [1]. A recent meta-analysis showed a better survival of BCS with whole breast irradiation compared to mastectomy [2]. The aim of BCS is to remove the breast cancer with a sufficient surrounding breast tissue to allow for both tumor-free resection margins, and achievement of an acceptable cosmetic result. The reported percentage of patients that receive one or more additional surgeries (re-operations) due to involved margins with invasive cancer or ductal carcinoma in situ (DCIS) after BCS varies, within a range of 1–37 % [[3], [4], [5], [6], [7]]. A re-operation may cause additional anxiety for the patient [8], impair the aesthetic outcome [9], and increase costs for the health system [10]. To improve intraoperative evaluation of the resected margins, several tools have been developed. Of these, intraoperative frozen section analysis has shown to be the most accurate [7,11], however it is a resource demanding method and requires immediate assistance from a pathologist. At our department, we use a single X-ray image of the surgical specimen as the standard imaging method for intraoperative margin assessment followed by a post-operative gross assessment of the specimen. We have recently conducted a meta-analysis where X-ray of the breast specimen was the most frequently reported radiological method for intraoperative margin assessment [12], but with a lower diagnostic accuracy in predicting histopathological margin status of 50–69 %, compared to other radiological modalities: Ultrasound 71–87 %, [4,[13], [14], [15]]; micro-CT 62–80 % [[16], [17], [18], [19]], and digital breast tomosynthesis (DBT) of 67–83 %, [12,[20], [21], [22], [23], [24]]. The DBT creates multiple 1 mm images reconstructed to a series of 2D images that gives a 3D impression of the whole breast specimen. This reduces the risk of breast tissue superimposition with a clearer depiction of the resected margins. We hypothesized that DBT gives a better view of all resected margins during BCS than a single X-ray image resulting in less patients with involved histopathological margins that require a re-operation.

We aimed to evaluate the number of re-operations due to involved resection margins after BCS in a randomized setting, comparing use of DBT with X-ray for intraoperative margin evaluation. Furthermore, we assessed the diagnostic accuracy of these methods to predict histopathological margin status. Finally, we evaluated potential clinical-pathological factors associated with increased risk of a re-operation.

## 2 Material and methods

### 2.1 Study population

The study was registered at ClinicalTrials.gov (NCT04478669), approved by the Ethics Committee for Central Region Denmark, and the Danish Data Protection Agency prior to inclusion and reported according to the consolidated standards of reporting trials (CONSORT) [25].

### 2.2 Study design

We conducted a parallel, randomized, non-blinded, controlled study, where patients were randomized 1:1 to intraoperative DBT (intervention) or X-ray (standard method) of their breast specimen. The randomization was performed in the Research Electronic Data Capture system (REDCap) [26]. The random allocation sequences were generated a priori by the independent trial REDCap administrator at Aarhus University. The allocation result was reported in the medical record by the first author, prior to initial BCS.

### 2.3 Eligibility criteria

**Fig. 1:**
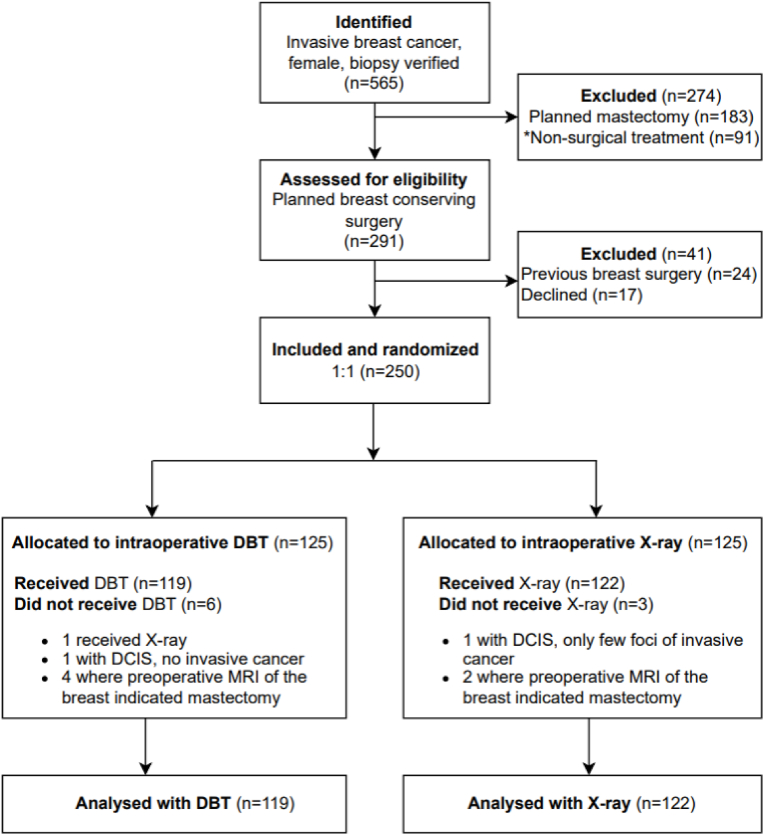
**Flowchart of the included study patients according to the consolidated standards of reporting trial (CONSORT)** Abbreviations: n, number of patients; DCIS, ductal carcinoma in situ; DBT, digital breast tomosynthesis; MRI, magnetic resonance imaging. *Non-surgical treatment: Breast cancer patients with distant metastases that received chemotherapy and patients that received hormone therapy only.

We included women, age ≥18 years, with biopsy-verified, invasive breast cancer planned for BCS. Inclusion of the first patient was September 17th, 2020, and last patient included, January 28th, 2022, at the Department of Plastic and Breast Surgery, Aarhus University Hospital. Patients planned for neoadjuvant chemotherapy (NACT) were included after completion of their systemic treatment. Patients with previous breast surgery, ductal carcinoma in situ (DCIS) with no invasive cancer, and patients planned for mastectomy were excluded (Fig. 1).

### 2.4 Perioperative data collection

Preoperative sonographic tumor size, mammographic breast density (categorized A-D, A: Almost entirely fatty and D: Extreme dense breast tissue) [27], age, menopausal status, NACT-status, tumor palpability, surgical outcomes (number and type of re-operations), histological tumor size, malignancy grade, histological subtype, DCIS outside the tumor (in the surrounding tissue at a distance from the tumor), human epidermal growth factor 2 (HER-2), estrogen receptor (ER), and lymph node status were collected from medical records and registered in REDCap.

### 2.5 Preoperative marking of the tumor

Patients with a non-palpable breast tumor or where the tumor was not definitely palpable, and palpable tumors of patients planned for NACT had the lesion marked with a coil guided by ultrasound, performed by the breast radiologist and its location visualized on a mammography.

In the morning, prior to surgery, the location of the coil was visualized on mammography and ultrasound to ensure its location and marked with ink on the skin by the breast radiologist. The surgeon used the ink on the patient's skin in combination with ultrasound as guidance for excision of the breast tumor.

### 2.6 Intraoperative imaging

**Fig. 2:**
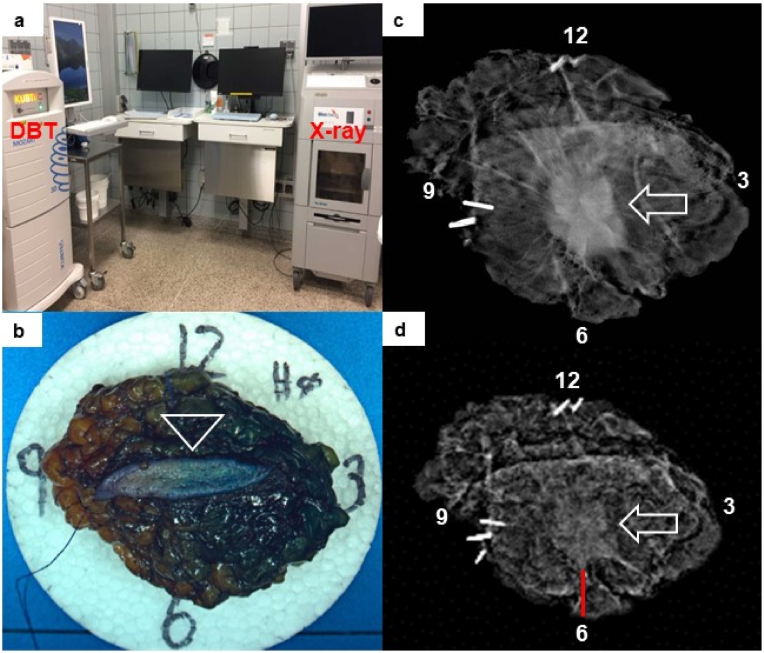
Intraoperative digital breast tomosynthesis of a breast cancer specimen with a true negative finding. a) The operating theater with the digital breast tomosynthesis (DBT), (intervention method) and the X-ray device (standard method). b) Breast specimen from a patient with invasive ductal breast cancer with a true negative finding. c) A sectional DBT-image through the middle and d) bottom of the specimen shows a sufficient distance from the centrally located tumor (white arrow), to the resected margins of 6 mm at 6 o'clock (red line). The radiologist assessed the intraoperative margins as tumor free that was confirmed by the pathologist in the final histopathology. (For interpretation of the references to colour in this figure legend, the reader is referred to the Web version of this article.)

After excision of the breast tumor, the surgeon marked the specimen with sutures and clips for orientation and placed it on a styrofoam bed, oriented clockwise and inserted in either the DBT or the X-ray device located in the operating theater (Fig. 2 a), as according to randomization. The Mozart® Specimen Tomosynthesis Imaging System, (KUBTEC Medical Imaging, Stratford, CT, USA) was used for the DBT images and the Faxitron™ intraoperative specimen digital radiography system (Hologic® Inc., Bedford, MA, USA) was used for the X-ray images. The images were sent digitally and analyzed by dedicated breast radiologists, who subsequently communicated their conclusion on margin status to the surgeon over the telephone. Based on the conclusion of the radiologist, a re-resection was performed in case of involvement with tumor or microcalcifications close to or in the resected margins. The surgeon then attached the re-resection to the main specimen before it was sent to pathological examination. Weight of the specimen and total analysis time was registered intraoperatively. Size and visibility of the tumor, overlying skin in the specimen, and if the resected margins were assessed as involved or tumor free was noted on a predefined sheet by the breast radiologists.

### 2.7 Postoperative histopathological analysis

As part of the routine diagnostics, breast pathologists assessed the specimen macro- and microscopically. An involved margin was defined as a margin width of 0 mm for invasive carcinoma and ≤2 mm for DCIS, and tumor free as > 0 mm for invasive cancer, and >2 mm for DCIS according to national and international guidelines [28,29]. The histopathological assessment of the resected margins was used as reference for the radiological assessment of the resected margins.

Conclusions from the radiologist based on the allocated imaging method were true positive (TP), if margins were truly involved by invasive cancer, DCIS, or both, and true negative (TN) if margins were tumor free on imaging as well as on final histopathology. False positive (FP) findings corresponded to margins assessed as involved by imaging but microscopically tumor free and false negative (FN), if considered tumor free on imaging but involved on final histopathology.

### 2.8 Statistical analysis

All analyses were performed in Stata 17 (StataCorp, 2021, college station, Texas, USA) with a significance level of 0.05. Prior to inclusion, a sample size calculation showed that 250 patients were needed to detect a reduction of involved margins from 20 % to 7 % between the DBT and X-ray group with a statistical power of 80 %. Patient characteristics were examined for differences between groups with *t*-test of the means for continuous variables after log-transformation when appropriate. Pearson's chi-squared test and Fisher's exact test (expected count <5) were used for categorical variables. Logistic regressions and ordinal logistic regressions were used to assess differences of the categorical variables between the groups with and without adjustment for possible confounders. Sensitivity, specificity, positive predictive value (PPV), negative predictive value (NPV), and accuracy of DBT and X-ray were calculated from raw diagnostic accuracy data. Significant risk factors for re-operation were examined by logistic regressions with and without adjustment for confounders, and with Bonferroni correction for multiple testing in the pairwise comparisons of breast density categories.

## 3 Results

### 3.1 Study demographics

A total of 250 invasive breast cancer patients were included and randomized. Nine out of 250 patients (6 with DBT and 3 with X-ray) were excluded due to eligibility criteria (Fig. 1). Subsequent analysis encompassed 241 breast specimens from 241 breast cancer patients, 119 with DBT (intervention) and 122 with standard X-ray. The perioperative patient characteristics between the DBT and X-ray groups were comparable (Table 1).

**Table 1:** Perioperative characteristics of all 241 invasive breast cancer patients.

	DBT, n (%)	X-ray, n (%)	Total, n (%)	*p*-value
Preoperative				

Number of patients	119	122	241	
Age [mean (min-max)]	62.9 (32–89)	65.5 (41–90)	64.2 (32–90)	0.06^a^
Menopausal status				
Premenopausal	23 (19.3)	17 (13.9)	40 (16.6)	0.26^b^
Postmenopausal	96 (80.7)	105 (86.1)	201 (83.4)	
Tumor size ultrasound, mm* [median (min-max)]				
≤ 20 (T1)	13 (5–40)	14 (3–40)	13 (3–40)	0.92^a^
> 20 (T2)	95 (80.5)	100 (82.0)	195 (81.3)	
	23 (19.5)	22 (18.0)	45 (18.8)	
Neoadjuvant chemotherapy				
Yes	8 (6.7)	11 (9.0)	19 (7.9)	0.51^b^
No	111 (93.3)	111 (91.0)	222 (92.1)	
Tumor palpable				
Palpable	66 (55.5)	61 (50.0)	127 (52.7)	0.40^b^
Non-palpable	53 (44.5)	61 (50.0)	114 (47.3)	
Breast density mammography				
A	18 (15.1)	22 (18.0)	40 (16.6)	0.88^c^
B	57 (47.9)	60 (49.2)	117 (48.5)	
C	38 (31.9)	34 (27.9)	72 (29.9)	
D	6 (5.0)	6 (4.9)	12 (5.0)	
**Postoperative**				
Tumor size histology, mm* [median (min-max)]				
≤ 20 (T1)	15 (4–37)	14 (0–40)	14 (0–40)	0.20^a^
> 20 (T2)	99 (83.2)	103 (84.4)	202 (83.8)	
	20 (16.8)	19 (15.6)	39 (16.2)	
Malignancy grade**				
I	34 (28.8)	27 (22.5)	61 (25.6)	0.48^b^
II	64 (54.2)	68 (56.7)	132 (55.5)	
III	20 (16.9)	25 (20.8)	45 (18.9)	
HER-2 status				
Negative	106 (89.1)	106 (86.9)	212 (88.0)	0.60^b^
Positive	13 (10.9)	16 (13.1)	29 (12.0)	
Estrogen receptor status				
0 %	10 (8.4)	11 (9.0)	21 (8.7)	0.87 ^b^
1–100 %	109 (91.6)	111 (91.0)	220 (91.3)	
Lymph node status				
Negative	87 (73.1)	88 (72.1)	175 (72.6)	0.86^b^
Positive	32 (26.9)	34 (27.9)	66 (27.4)	
Histological subtypes				
Invasive ductal	90 (75.6)	96 (78.7)	186 (77.2)	0.65^c^
Lobular	18 (15.1)	13 (10.7)	31 (12.9)	
Invasive ductal and lobular	5 (4.2)	4 (3.3)	9 (3.7)	
Special types***	6 (5.0)	9 (7.4)	15 (6.2)	

Abbreviations: DBT, digital breast tomosynthesis; n, number of patients; HER-2, human epidermal growth factor 2; T1 and T2, tumor stage; a) *t*-test; b) Pearson's chi-squared test; c) Fischer's exact test. *There was one missing allocated to DBT, where the tumor was not visible on preoperative ultrasound. A *t*-test was performed on log transformed data for preoperative tumor size ultrasound and on log(y+1) for postoperative tumor size histology, where two patients from the X-ray group had a tumor size 0 mm after neoadjuvant chemotherapy. **Malignancy grade data was missing in 3 patients that received neoadjuvant chemotherapy, one in the DBT- and two in the X-ray group. ***Special types of carcinoma (squamous, micropapillary, apocrine breast carcinoma and one no further specified).

### 3.2 Final histopathological margin status and surgical outcomes

There was no difference in the percentage of patients with involved resection margins on final histopathology, when comparing the DBT 21/119 (17.6 %) and X-ray cohorts 25/122 (20.5 %), in neither non-adjusted analysis (*p* = 0.57) or when adjusted for possible confounders such as NACT, age, and preoperative mammographic breast density (*p* = 0.49) (Table 2). DCIS was the main cause of involved resection margins microscopically in 12/119 (10.1 %) of the cases with DBT and in 13/122 (10.7 %) of the cases with X-ray (Table 2).

**Table 2:** Final histopathological margin status and surgical outcomes.

Number of patients	DBT	X-ray	Total	*p*-value	*p*-value
	n (%)	n (%)	n (%)	non-adjusted	adjusted*
	119	122	241		
Histological margin status					
Tumor free margins	98 (82.4)	97 (79.5)	195 (80.9)	0.57^a^	0.49^a^
Involved margins	21 (17.6)	25 (20.5)	46 (19.1)		
Invasive	7 (5.9)	7 (5.7)	14 (5.8)		
DCIS	12 (10.1)	13 (10.7)	25 (10.4)		
Invasive and DCIS	2 (1.7)	5 (4.1)	7 (2.9)		
Intraoperative re-resections					
guided by imaging**					
No	70 (63.1)	78 (70.9)	148 (67.0)	0.21^a^	0.23^a^
Yes	41 (36.9)	32 (29.1)	73 (33.0)		
Re-operation					
No	99 (83.2)	98 (80.3)	197 (81.7)	0.56^a^	0.57^a^
Yes	20 (16.8)	24 (19.7)	44 (18.3)		
Breast conserving	17 (14.3)	22 (18.0)	39 (16.2)		
Mastectomy	3 (2.5)	2 (1.6)	5 (2.1)		
Number of re-operations					
0	99 (83.2)	98 (80.3)	197 (81.7)	0.69^b^	0.77^b^
1	11 (9.2)	19 (15.6)	30 (12.4)		
2	5 (4.2)	4 (3.3)	9 (3.7)		
3	4 (3.4)	1 (0.8)	5 (2.1)		

Abbreviations: DBT, digital breast tomosynthesis; n, number; DCIS, ductal carcinoma in situ; *p-value adjusted for neoadjuvant chemotherapy, age and breast density on preoperative mammography; a) logistic regression and b) ordinal logistic regression. Intraoperative re-resection of the operating cavity was guided by the intraoperative imaging method, DBT or X-ray of the specimen. **there were 20 missing data (8 allocated to DBT and 12 to X-ray) where the resected margins could not be assessed on imaging.

In 2/195 (1.0 %) of the cases assessed as tumor free on final histopathology (Suppl. Table 1) the patients nevertheless received a re-operation. In one case the coil was lost during primary BCS and in the other case the coil in a satellite of the index tumor was not found in the final histopathology. In 4/46 (8.7 %) of the cases, the superficial margin was involved with DCIS in the histopathology, but the surgeons considered the lesion was radically removed since the dissection was in the subcutaneous fascia.

There was no difference in the proportion of patients that received at least one re-operation between the DBT and X-ray cohorts, 16.8 % vs 19.7 %, in the adjusted analysis (*p* = 0.57) (Table 2).

The median total analysis time was significantly longer (*p* < 0.001) using intraoperative DBT (10 min) compared to X-ray (6 min) in the adjusted analysis. There was no difference in the median weight of the resected breast specimen (*p* = 0.57) in the adjusted analysis (Suppl. Table 2).

### 3.3 Diagnostic accuracy of DBT and X-ray to predict histopathological margin status

The diagnostic performance of DBT and X-ray to predict histopathological margin status is shown in Table 3. Although the sensitivity, specificity, PPV, and NPV tended to be better for DBT than for X-ray, the confidence intervals showed appreciable overlap when comparing the groups.

**Table 3:** Diagnostic accuracy of intraoperative digital breast tomosynthesis and X-ray of the main breast specimen to predict histopathological resection margin status.

	Pathology Involved: n	Pathology Tumor free: n	Total n
**DBT:** Involved n	TP: 24	FP: 17	41
**DBT:** Tumor free n	FN: 8	TN: 62	70
**Total**: n	32	79	111*
Sensitivity: 75.0 %, CI [56.6; 88.5]			
Specificity: 78.5 %, CI [67.8; 86.9]			
PPV: 58.5 %, CI [42.1; 73.7]			
NPV: 88.6 %, CI [78.7; 94.9]			
Accuracy: 77.5 %, CI [68.6; 84.9]			
	**Pathology** **Involved: n**	**Pathology** **Tumor free: n**	**Total n**
**X-ray:** Involved n	TP: 10	FP: 22	32
**X-ray:** Tumor free n	FN: 14	TN: 64	78
**Total:** n	24	86	110**
Sensitivity: 41.7 %, CI [22.1; 63.4]			
Specificity: 74.4 %, CI [63.9; 83.2]			
PPV: 31.3 %, CI [16.1; 50.0]			
NPV: 82.1 %, CI [71.7; 89.8]			
Accuracy: 67.3 % CI [57.7; 75.9]			

Abbreviations: n, number of specimens; DBT, digital breast tomosynthesis; TP, true positive; FP, false positive; FN, false negative; TN, true negative; CI, 95 % confidence interval; PPV, positive predictive value; NPV, negative predictive Value. n, total number of specimens that was assessed by the radiologist with the allocated imaging method.*There were 8 missing out of 119 specimens allocated to DBT and **12 out of 122 specimens allocated to the X-ray method, where the resected margins could not be assessed on imaging.

The diagnostic accuracy to predict histopathological margin status was 77.5 % (95 % CI: 68.6–84.9) for DBT and 67.3 (95 % CI: 57.7–75.9) for X-ray.

**Fig. 3:**
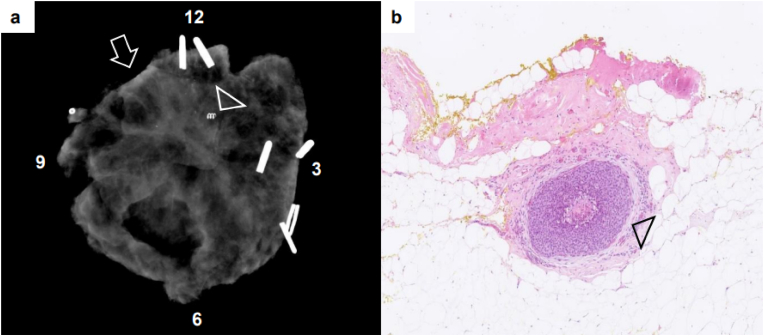
Intraoperative X-ray image of a breast cancer specimen with a true positive finding. a) An X-ray image from a patient with an invasive ductal cancer in the left breast with a true positive finding. The tumor was non-palpable and coil-marked by the radiologist preoperatively (arrowhead). The radiologist concluded the resection margin at 11 o'clock was involved with tumor and microcalcifications (arrow). The surgeon made an intraoperative re-resection at 9–12 o'clock. b) The finding was confirmed on final histopathology with ductal carcinoma in situ (arrowhead), outside the invasive tumor area, less than 2 mm from the re-resection margin (yellow ink) (Magnification × 50). (For interpretation of the references to colour in this figure legend, the reader is referred to the Web version of this article.)

**Fig. 4:**
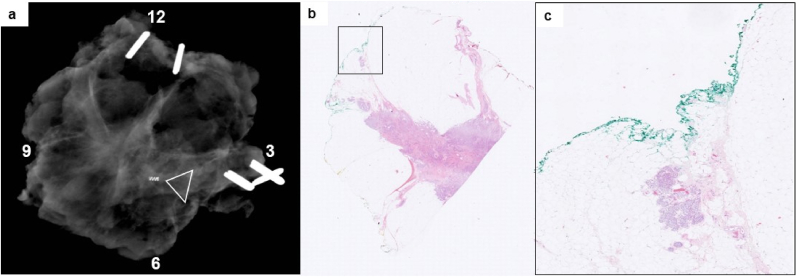
Intraoperative X-ray image of a breast cancer specimen with a false negative finding. a) An X-ray image of a breast specimen from a patient with a non-palpable, combined invasive ductal and lobular breast cancer with a false negative finding. The tumor was marked with a coil by the radiologist preoperatively (arrowhead). The radiologist concluded that the resection margins where tumor free with no microcalcifications. b) On microscopy, ductal carcinoma in situ was present <1 mm from the resected margins at 6 to 12 o'clock (green ink) (Magnification × 10). c) Inset of the involved resection margins from image b) (Magnification × 38). (For interpretation of the references to colour in this figure legend, the reader is referred to the Web version of this article.)

An illustration of a breast specimen with a TN finding on DBT is shown in (Fig. 2 b-d) and illustrated in a 2D video of the whole specimen (suppl. data). A case with a TP finding on X-ray is shown in Fig. 3 and with a FN finding on X-ray in Fig. 4. In the latter case, the radiologist assessed the intraoperative margins as tumor free, and the pathologist found it involved with DCIS in the final histopathology. The patient later received two re-operations, an additional BCS and mastectomy as a third and final surgical procedure, due to continuous DCIS involvement of the resection margins.

In 20 patients, the resected margins were difficult to assess on intraoperative imaging and the margins could not be evaluated, 8 in the DBT and 12 in the X-ray group, *p* = 0.38 (Suppl. Table 3). In 9 cases the patient had high mammographic breast density, in three cases the tumor border was difficult to differentiate from the surrounding fibro glandular breast tissue, in one case only a small rim of the tumor was left in the periphery of a collapsed cystic component, and in one case there was a technical problem with the imaging. There were 6 cases after NACT, three of which had complete response on breast magnetic resonance imaging (MRI) and three cases with residual tumor visible on breast MRI after NACT, where only the coil was visible on intraoperative X-ray.

### 3.4 Risk factors associated with at least one re-operation

We identified 5 risk factors for receiving one or more re-operation (Table 4) in the non-adjusted analysis. After adjusting for age, NACT, and grade of mammographic breast density, patients with DCIS outside the tumor showed the highest OR of 9.4 (95 % CI: 4.3–20.6, *p* < 0.001) (Table 5). Other factors associated with a risk of receiving at least one re-operation were: High, type D mammographic breast density (OR = 6.1, 95 % CI: 1.0–38.1, *p* = 0.047), non-evaluable tumor margins in the breast specimen on intraoperative imaging with DBT or X-ray (OR = 3.8, 95 % CI: 1.3–10.8, *p* = 0.016), NACT (OR = 3.0, 95 % CI: 1.0–8.8, *p* = 0.048), and large (T2) vs small (T1) tumor size on ultrasound (OR = 2.6, 95 % CI: 1.0–6.4, *p* = 0.045).

**Table 4:** Risk factors for receiving ≥1 re-operation for all 241 patients in the non-adjusted analysis.

	Re-operation	Re-operation	Total	*p*-value
	Yes, n (%)	No, n (%)	(%)	
Preoperative				

Number of patients	44	197	241	
Tumor size ultrasound, mm*				
≤ 20 (T1)	27 (62.8)	168 (85.3)	195 (81.3)	**0.001**^a^
> 20 (T2)	16 (37.2)	29 (14.7)	45 (18.8)	
Neoadjuvant chemotherapy				
Yes	8 (18.2)	11 (5.6)	19 (7.9)	**0.010**^b^
No	36 (81.8)	186 (94.4)	222 (92.1)	
Tumor palpable				
Yes	27 (61.4)	100 (50.8)	127 (52.7)	0.20^a^
No	17 (38.6)	97 (49.2)	114 (47.3)	
Breast density mammography				
A	6 (13.6)	34 (17.3)	40 (16.6)	
B	18 (40.9)	99 (50.3)	117 (48.5)	**0.011**^b^
C	13 (29.5)	59 (29.9)	72 (29.9)	
D	7 (15.9)	5 (2.5)	12 (5.0)	
Malignancy grade**				
I	7 (16.3)	54 (27.7)	61 (25.6)	0.30^a^
II	27 (62.8)	105 (53.8)	132 (55.5)	
III	9 (20.9)	36 (18.5)	45 (18.9)	
**Intraoperative specimen imaging**				
Method for margin assessment				
DBT	20 (45.5)	99 (50.3)	119 (49.4)	0.56^a^
X-ray	24 (54.5)	98 (49.7)	122 (50.6)	
Microcalcifications in specimen				
Yes	9 (20.5)	32 (16.2)	41 (17.0)	0.50^a^
No	35 (79.5)	165 (83.8)	200 (83.0)	
Overlaying skin in specimen***				
Yes	8 (20.5)	29 (16.6)	37 (17.3)	0.56^a^
No	31 (79.5)	146 (83.4)	177 (82.7)	
Evaluable on imaging				
Yes	34 (77.3)	187 (94.9)	221 (91.7)	**< 0.001**^b^
No	10 (22.7)	10 (5.1)	20 (8.3)	
**Postoperative**				
Tumor size histology, mm				
≤ 20 (T1)	33 (75.0)	169 (85.8)	202 (83.8)	0.08^a^
> 20, (T2)	11 (25.0)	28 (14.2)	39 (16.2)	
DCIS outside tumor, histology****				
Yes	25 (58.1)	28 (14.4)	53 (22.3)	< **0.001**^a^
No	18 (41.9)	167 (85.6)	185 (77.7)	
HER-2 status				
Positive	8 (18.2)	21 (10.7)	29 (12.0)	0.17^a^
Negative	36 (81.8)	176 (89.3)	212 (88.0)	
Estrogen receptor status				
0 %	3 (6.8)	18 (9.1)	21(8.7)	0.77^b^
1–100 %	41 (93.2)	179 (90.9)	220 (91.3)	
Lymph node status				
Positive	10 (22.7)	56 (28.4)	66 (27.4)	
Negative	34 (77.3)	141 (71.6)	175 (72.6)	0.44^a^

Abbreviations: Number of patients (n). Digital breast tomosynthesis (DBT). Ductal carcinoma in situ (DCIS). Human epidermal growth factor receptor-2 (HER-2) status. ^a^Pearson's chi-squared test, ^b^Fisher's exact test. *One missing data with re-operation where the tumor was not visible on pre-operative ultrasound. **Missing data for 3 study patients: 1 with and 2 with no re-operation. ***In 27 study patients: 22 with no re-operation and 5 with a re-operation; the radiologist could not assess on imaging, if there was any overlying skin in the specimen. ****Missing data for 3 study patients: 1 with re-operation and 2 with no re-operation.

**Table 5:** Risk of ≥1 re-operation in the *non-adjusted and **adjusted analysis for all 241 patients.

	OR*	95 % CI*	*p*-value*	OR**	95 % CI**	*p*-value**
**Preoperative**						
NACT: Yes vs No	3.8	1.4–10.0	**0.011**	3.0	1.0–8.8	**0.048**
Tumor size						
ultrasound, mm						
>20 (T2) vs ≤ 20 (T1)	3.4	1.6–7.1	**0.001**	2.6	1.0–6.4	**0.045**
Breast density mammography, high vs low***						
D vs A	7.9	1.4–46	**0.011**	6.1	1.0–38.1	**0.047**
C vs A	1.2	0.34–4.5	>0.99	1.1	0.29–4.1	>0.99
B vs A	1.0	0.30–3.5	>0.99	1.0	0.28–3.3	>0.99
**Intraoperative**						
Evaluable on imaging with						
DBT or X-ray						
No vs Yes	5.5	2.1–14.2	**< 0.001**	3.8	1.3–10.8	**0.016**
**Postoperative**						
DCIS outside tumor, histology						
Yes vs No	8.3	4.0–17.1	**< 0.001**	9.4	4.3–20.6	**< 0.001**

Abbreviations: NACT, neoadjuvant chemotherapy; OR, odds ratio; CI, confidence interval; DBT, digital breast tomosynthesis; T1 and T2, Tumor stage. *Non adjusted logistic regression analysis. **Logistic regression analysis adjusted for NACT, age and preoperative breast density on mammography. ***Comparisons (CI and *p*-value) against group A are adjusted for multiple testing by Bonferroni correction for 3 tests.

## 4 Discussion

Involved resection margins after BCS remains a clinical challenge, which most often requires a re-operation to ensure adequate removal [30,31] and to reduce the risk of local recurrence [32,33].

We found no difference in the percentage of patients with re-operation due to involved margins after BCS comparing the use of intraoperative DBT and X-ray. Previously, two retrospective studies have evaluated the impact of intraoperative DBT and X-ray on the number of involved resection margins and re-operations for patients planned for BCS, with divergent results. In concordance with our study, the most recent retrospective study with 228 invasive breast cancer patients found no difference in the percentage of patients with involved resection margins between DBT 15.8 % and X-ray method 23.9 % (*p* = 0.22) [34]. A second retrospective study, where 191 received DBT and 466 X-ray, concluded that intraoperative DBT reduced the proportion of re-operations to 5 % as compared to 11 % with X-ray (p = 0.02) [35]. However, the groups were not fully comparable in that study, since the X-ray group had more patients with DCIS, which in previous reports have been associated with an increased risk of involved histopathological margins [36,37] and thus will result in a higher proportion of re-operations.

In the present study, no significant difference was found in the diagnostic accuracy of intraoperative DBT and X-ray to predict histopathological margin status. Our findings contrast with two prospective studies that found a higher accuracy in predicting histopathological margin status for DBT of (82%–92 %) compared to X-ray (65%–79 %) [23,24]. However, these studies did not explore if intraoperative DBT versus X-ray had an impact on the percentage of patients with involved histopathological margins and their surgical outcomes.

We identified 5 risk factors for receiving at least one re-operation, where DCIS outside the tumor was the strongest predictor for a re-operation in consensus with two previous, large studies [33,38]. The relative high percentage of re-operations of 18 % in our study can partly be explained by DCIS outside the tumor that can be difficult to visualize with both DBT and X-ray, as the suspected microcalcifications that represents DCIS are not present in all the cases [40]. Furthermore, it can be difficult to differentiate between benign and suspected microcalcifications as in DCIS [16].

In most of the non-evaluable cases, the patients had a high breast density, where the radiologist could not assess the tumor margins, compromising the distinction between neoplastic and benign breast tissue. In accordance with the literature, we found that patients with extremely high mammographic breast density, after NACT, and a large (T2) tumor size have a high risk for receiving a re-operation after BCS [[38], [39], [40]].

The total analysis time of DBT in our study was 4 min longer than of X-ray. A previous report found a prolonged total surgical time of 26 min [34]. Although the authors did not measure the total analysis time of each method, they suggested that the prolonged surgery time was due to a longer analysis time analyzing multiple images with DBT compared to a single X-ray image as in accordance with our findings.

In our study, DBT did not improve intraoperative margin assessment compared to X-ray. However, other promising intraoperative methods have arrived on the scene in the meantime. One of these is ultra-high-resolution optical coherence tomography (UHR-OCT) combined with deep learning with a sensitivity of 93 % and a specificity of 95 % to predict histopathological margin status [41]. The UHR-OCT seems to be able to differentiate cancer cells from fibro glandular and fatty tissue to a depth of 2 mm [42]. Another method, intraoperative MRI of the specimen reported a sensitivity and a specificity to predict histopathological margin status of 80 % and 84 % [43]. In a randomized controlled trial, use of cavity shaved margins surgical technique reduced the percentage of re-operations from 21 % to 10 % (*p* = 0.02) [44]. This method is, however, associated with a great variation in the size of the shaved cavity samples. Finally, using the pathological assisted intraoperative gross evaluation of the specimen, combining both palpation and inspection of the fresh breast specimen showed a sensitivity of 49 %, specificity of 86 % to predict histopathological margin status [45]. Despite the various promising methods, an intraoperative method that can better visualize the DCIS in the resected margins is still needed.

This is the first relatively large, randomized study to evaluate the proportion of involved histopathological margins and re-operations comparing intraoperative DBT with X-ray of the breast specimen. The study has some limitations. To comply with the routine diagnostic workflow at our departments, blinding of the surgeons and the radiologists was not an option, as well as evaluating the interobserver variability between the different breast radiologists. However, we do not believe that these limitations affected the proportion of involved histopathological margins and surgical outcomes in our study.

In conclusion, we found no difference in the number of re-operations or in diagnostic accuracy to predict histopathological margin status comparing intraoperative DBT with X-ray for intraoperative margin evaluation. DCIS outside the tumor carry the highest risk for receiving at least one re-operation. Intraoperative imaging methods that can better visualize DCIS are needed to obtain tumor free resection margins in BCS.

## Source of funding

This work was supported by following funds: 10.13039/501100009708Novo Nordisk Foundation [NNF19OC0057928]; 10.13039/100010809Jascha Fonden [7721], Denmark; 10.13039/501100005860Helsefonden [19-B-0017], Denmark; Riisfort Fonden, Denmark; 10.13039/100008363Danish Cancer Society [R231-A13754], Denmark and Louis-Hansen's Fonden [19-2B-5418], Denmark. The funds of this study were not involved in the design of the study, collection, management, or data analysis, in the interpretation of the results, the preparation of the manuscript or in the decision to submit the article for publication.

## CRediT authorship contribution statement

**Irina Palimaru Manhoobi:** Data curation, Formal analysis, Funding acquisition, Investigation, Methodology, Project administration, Writing - original draft, Writing - review & editing. **Trine Tramm:** Methodology, Supervision, Validation, Writing - review & editing. **Søren Redsted:** Methodology, Supervision, Validation, Writing - review & editing. **Anne Bodilsen:** Formal analysis, Supervision, Validation, Writing - review & editing. **Leslie Foldager:** Formal analysis, Supervision, Validation, Writing - review & editing. **Peer Christiansen:** Conceptualization, Funding acquisition, Investigation, Methodology, Project administration, Resources, Supervision, Writing - review & editing.

## Appendix A Supplementary data

Multimedia component 1 Supplementary tables

Multimedia component 2 Supplementary video of a specimen with digital breast tomosynthesis

## References

[1] Fisher B., Anderson S., Bryant J., Margolese R.G., Deutsch M., Fisher E.R. (2002). Twenty-year Follow-up of a randomized trial comparing total mastectomy, Lumpectomy, and Lumpectomy plus irradiation for the treatment of invasive breast cancer. N Engl J Med.

[2] Christiansen P., Mele M., Bodilsen A., Rocco N., Zachariae R. (2022). Breast-conserving surgery or mastectomy?. Annals of Surgery Open.

[3] McCahill L.E., Single R.M., Aiello Bowles E.J., Feigelson H.S., James T.A., Barney T. (2012). Variability in reexcision following breast conservation surgery. JAMA, J Am Med Assoc.

[4] Ramos M., Díaz J.C., Ramos T., Ruano R., Aparicio M., Sancho M. (2013). Ultrasound-guided excision combined with intraoperative assessment of gross macroscopic margins decreases the rate of reoperations for non-palpable invasive breast cancer. Breast.

[5] McCormick J.T., Keleher A.J., Tikhomirov V.B., Budway R.J., Caushaj P.F. (2004). Analysis of the use of specimen mammography in breast conservation therapy. Am J Surg.

[6] Weber W.P., Engelberger S., Viehl C.T., Zanetti-Dallenbach R., Kuster S., Dirnhofer S. (2008). Accuracy of frozen section analysis versus specimen radiography during breast-conserving surgery for nonpalpable lesions. World J Surg.

[7] Racz J.M., Glasgow A.E., Keeney G.L., Degnim A.C., Hieken T.J., Jakub J.W. (2020). Intraoperative pathologic margin analysis and Re-excision to Minimize reoperation for patients undergoing breast-conserving surgery. Ann Surg Oncol.

[8] Amiel C.R., Fisher H.M., Carver C.S., Antoni M.H. (2016). The importance of stress management among postresection breast cancer patients. Future Oncol.

[9] Heil J., Breitkreuz K., Golatta M., Czink E., Dahlkamp J., Rom J. (2012). Do reexcisions impair aesthetic outcome in breast conservation surgery? Exploratory analysis of a prospective cohort study. Ann Surg Oncol.

[10] Grant Y., Al-Khudairi R., St John E., Barschkett M., Cunningham D., Al-Mufti R. (2019). Patient-level costs in margin re-excision for breast-conserving surgery. Br J Surg.

[11] St John E.R., Al-Khudairi R., Ashrafian H., Athanasiou T., Takats Z., Hadjiminas D.J. (2017). Diagnostic accuracy of intraoperative techniques for margin assessment in breast cancer surgery a meta-analysis. Ann Surg.

[12] Manhoobi I.P., Bodilsen A., Nijkamp J., Pareek A., Tramm T., Redsted S. (2022). Diagnostic accuracy of radiography, digital breast tomosynthesis, micro-CT and ultrasound for margin assessment during breast surgery: a systematic review and meta-analysis. Acad Radiol.

[13] Pop M.M., Cristian S., Hanko-Bauer O., Ghiga D.V., Georgescu R. (2018). Obtaining adequate surgical margin status in breast-conservation therapy: intraoperative ultrasound-guided resection versus specimen mammography. Clujul Med.

[14] Mesurolle B., El-Khoury M., Hori D., Phancao J.P., Kary S., Kao E. (2006). Sonography of postexcision specimens of nonpalpable breast lesions: value, limitations, and description of a method. Am J Roentgenol.

[15] Moschetta M., Telegrafo M., Introna T., Coi L., Rella L., Ranieri V. (2015). Role of specimen us for predicting resection margin status in breast conserving therapy. Giornale Di Chirurgia.

[16] Janssen N.N.Y., van Seijen M., Loo C.E., Vrancken Peeters M.J.T.F.D., Hankel T., Sonke J.J. (2019). Feasibility of micro–computed tomography imaging for direct assessment of surgical resection margins during breast-conserving surgery. J Surg Res.

[17] McClatchy D.M., Zuurbier R.A., Wells W.A., Paulsen K.D., Pogue B.W. (2018). Micro-computed tomography enables rapid surgical margin assessment during breast conserving surgery (BCS): correlation of whole BCS micro-CT readings to final histopathology. Breast Cancer Res Treat.

[18] Qiu S.Q., Dorrius M.D., de Jongh S.J., Jansen L., de Vries J., Schröder C.P. (2018). Micro-computed tomography (micro-CT) for intraoperative surgical margin assessment of breast cancer: a feasibility study in breast conserving surgery. Eur J Surg Oncol.

[19] DiCorpo D., Tiwari A., Tang R., Griffin M., Aftreth O., Bautista P. (2020). The role of Micro-CT in imaging breast cancer specimens. Breast Cancer Res Treat.

[20] Mario J., Venkataraman S., Fein-Zachary V., Knox M., Brook A., Slanetz P. (2019). Lumpectomy specimen radiography: does orientation or 3-dimensional tomosynthesis improve margin assessment?. Can Assoc Radiol J.

[21] Park K.U., Kuerer H.M., Rauch G.M., Leung J.W.T., Sahin A.A., Wei W. (2019). Digital breast tomosynthesis for intraoperative margin assessment during breast-conserving surgery. Ann Surg Oncol.

[22] Chand J.T., Sharma M.M., Dharmarajan J.P., Nambiar A. (2019). Digital breast tomosynthesis as a tool in confirming negative surgical margins in non-palpable breast lesions. Indian J Surg Oncol.

[23] Romanucci G., Mercogliano S., Carucci E., Cina A., Zantedeschi E., Caneva A. (2021). Diagnostic accuracy of resection margin in specimen radiography: digital breast tomosynthesis versus full-field digital mammography. Radiologia Medica.

[24] Garlaschi A., Fregatti P., Oddone C., Friedman D., Houssami N., Calabrese M. (2019). Intraoperative digital breast tomosynthesis using a dedicated device is more accurate than standard intraoperative mammography for identifying positive margins. Clin Radiol.

[25] Schulz K.F., Altman D.G., Moher D. (2010). CONSORT 2010 statement: updated guidelines for reporting parallel group randomised trials. J Pharmacol Pharmacother.

[26] Harris P.A., Taylor R., Minor B.L., Elliott V., Fernandez M., O'Neal L. (2019). The REDCap consortium: building an international community of software platform partners. J Biomed Inf.

[27] Spak D.A., Plaxco J.S., Santiago L., Dryden M.J., Dogan B.E. (2017). BI-RADS® fifth edition: a summary of changes. Diagn Interv Imaging.

[28] Moran M.S., Schnitt S.J., Giuliano A.E., Harris J.R., Khan S.A., Horton J. (2014). Society of surgical oncology-American Society for Radiation Oncology consensus guideline on margins for breast-conserving surgery with whole-breast irradiation in stages i and II invasive breast cancer. Ann Surg Oncol.

[29] Morrow M., Zee KJ Van, Solin L.J., Houssami N., Chavez-Macgregor M., Harris J.R. (2016). Society of surgical oncology-American society for radiation oncology-American society of clinical oncology consensus guideline on margins for breast-conserving surgery with whole-breast irradiation in ductal carcinoma in situ. Oncol.

[30] Aziz D., Rawlinson E., Narod S.A., Sun P., Lickley H.L.A., McCready D.R. (2006). The role of reexcision for positive margins in optimizing local disease control after breast-conserving surgery for cancer. Breast J.

[31] Smitt M.C., Nowels K., Carlson R.W., Jeffrey S.S. (2003). Predictors of reexcision findings and recurrence after breast conservation. Int J Radiat Oncol Biol Phys.

[32] Offersen B.V., Alsner J., Nielsen H.M., Jakobsen E.H., Nielsen M.H., Krause M. (2020). Hypofractionated versus standard Fractionated radiotherapy in patients with early breast cancer or ductal carcinoma in situ in a randomized Phase III trial: the DBCG HYPO trial. J Clin Oncol.

[33] Bodilsen A., Bjerre K., Offersen B.V., Vahl P., Ejlertsen B., Overgaard J. (2015). The Influence of repeat surgery and residual disease on recurrence after breast-conserving surgery: a Danish breast cancer Cooperative group study. Ann Surg Oncol.

[34] Joel C., Ciampa M., O'Hara T., Bandera B.C., Mangieri C.W. (2023). Effect of three-dimensional intraoperative imaging on surgical outcomes with breast conservation therapy. Am J Surg.

[35] Partain N., Calvo C., Mokdad A., Colton A., Pouns K., Clifford E. (2020). Differences in Re-excision rates for breast-conserving surgery using intraoperative 2D versus 3D tomosynthesis specimen radiograph. Ann Surg Oncol.

[36] Pleijhuis R.G., Kwast A.B.G., Jansen L., de Vries J., Lanting R., Bart J. (2013). A validated web-based nomogram for predicting positive surgical margins following breast-conserving surgery as a preoperative tool for clinical decision-making. Breast.

[37] Ellbrant J., Gulis K., Plasgård E., Svensjö T., Bendahl P.O., Rydén L. (2021). Validated prediction model for positive resection margins in breast-conserving surgery based exclusively on preoperative data. BJS Open.

[38] van Deurzen C.H.M. (2016). Predictors of surgical margin following breast-conserving surgery: a large Population-based cohort study. Ann Surg Oncol.

[39] Walsh S.M., Brennan S.B., Zabor E.C., Rosenberger L.H., Stempel M., Lebron-Zapata L. (2019). Does breast density increase the risk of Re-excision for women with breast cancer having breast-conservation therapy?. Ann Surg Oncol.

[40] Volders J.H., Haloua M.H., Krekel N.M.A., Negenborn V.L., Barbé E., Sietses C. (2016). Neoadjuvant chemotherapy in breast-conserving surgery – Consequences on margin status and excision volumes: a nationwide pathology study. Eur J Surg Oncol.

[41] Bareja R., Mojahed D., Hibshoosh H., Hendon C. (2022). Classifying breast cancer in ultrahigh-resolution optical coherence tomography images using convolutional neural networks. Appl Opt.

[42] Duan Y., Guo D., Zhang X., Lan L., Meng H., Wang Y. (2023). Diagnostic accuracy of optical coherence tomography for margin assessment in breast-conserving surgery: a systematic review and meta-analysis. Photodiagnosis Photodyn Ther.

[43] Thill M., Szwarcfiter I., Kelling K., van Haasteren V., Kolka E., Noelke J. (2022). Magnetic resonance imaging system for intraoperative margin assessment for DCIS and invasive breast cancer using the ClearSight^TM^ system in breast-conserving surgery—results from a postmarketing study. J Surg Oncol.

[44] Chagpar A.B., Killelea B.K., Tsangaris T.N., Butler M., Stavris K., Li F. (2015). A randomized, controlled trial of cavity shave margins in breast cancer. N Engl J Med.

[45] Nunez A., Jones V., Schulz-Costello K., Schmolze D. (2020). Accuracy of gross intraoperative margin assessment for breast cancer: experience since the SSO-ASTRO margin consensus guidelines. Sci Rep.

