# Supplementary material for: Digital breast tomosynthesis versus X-ray of the breast specimen for intraoperative margin assessment: A randomized trial
Source: Breast. 2023 Dec 1;73:103616. doi: 10.1016/j.breast.2023.103616 (PMC10749898; doi:10.1016/j.breast.2023.103616)
Supplement: Multimedia component 1 [file mmc1.docx]

**Supplementary data**

**Supplementary Table 1:**

Final histopathological margin and re-operation status of all 241 study patients

|  | **Re-operation: Yes (%)** | **Re-operation: No (%)** | **Total** |
| --- | --- | --- | --- |
| Number of patients | 44 (18.3) | 197 (81.7) | 241 |
| Involved | 42 (91.3) | 4 (8.7) | 46 |
| Tumor free | 2 (1.0) | 193 (99.0) | 195 |

Involved resection margins in the final histopathology was either due to ductal carcinoma in situ (DCIS), invasive cancer or both (DCIS with invasive cancer).

**Supplementary Table 2**

Total analysis time and weight of the specimen with intraoperative DBT and X-ray of the specimen

|  | **DBT** | **X-ray** | **Total** | ***p*-value**  non-adjusted | ***p*-value**  adjusted |
| --- | --- | --- | --- | --- | --- |
| Number of patients | 119 | 122 | 241 |  |  |
| Total analysis time, min**  [median (min-max)] | 10 (2-32) | 6 (1-27) | 8 (1-32) | **< 0.001**^a^ | **< 0.001**^b^ |
| Weight of specimen, g***  [median (min-max)] | 34 (7-306) | 32 (7-148) | 33 (7-306) | 0.75^a^ | 0.57^b^ |

Abbreviations: N (number of patients); DBT, digital breast tomosynthesis. ^a^Negative binomial regression analysis non-adjusted and after ^b^adjustment for neoadjuvant chemotherapy, age, and preoperative breast density. **Missing data from 6 study participants. ***Missing data from 5 study participants.

**Supplementary Table 3**

Number of patients where the resected margins of the specimen were difficult to assess (non-evaluable on imaging) with DBT and X-ray

|  | **DBT, n (%)** | **X-ray, n (%)** | **Total, n (%)** | ***p*-value** |
| --- | --- | --- | --- | --- |
| Evaluable on imaging  Yes (%) | 111 (93.3) | 110 (90.2) | 221 (91.7) | 0.38^a^ |
| No (%) | 8 (6.7) | 12 (9.8) | 20 (8.3) |  |

^a^Pearson’s chi-squared test. Abbreviations: DBT, digital breast tomosynthesis.
